# How to Realize the Benefits of Point-of-Care Testing at the General Practice: A Comparison of Four High-Income Countries

**DOI:** 10.34172/ijhpm.2021.143

**Published:** 2021-10-13

**Authors:** Deon Lingervelder, Hendrik Koffijberg, Jon D. Emery, Paul Fennessy, Christopher P. Price, Harm van Marwijk, Torunn B. Eide, Sverre Sandberg, Jochen W.L. Cals, Joke T.M. Derksen, Ron Kusters, Maarten J. IJzerman

**Affiliations:** ^1^Health Technology and Services Research Department, Technical Medical Centre, University of Twente, Enschede, The Netherlands.; ^2^Department of General Practice and Centre for Cancer Research, University of Melbourne, Melbourne, VIC, Australia.; ^3^Department of Health & Human Services, State Government of Victoria, Melbourne, VIC, Australia.; ^4^Nuffield Department of Primary Care Health Sciences, University of Oxford, Oxford, UK.; ^5^Department of Primary Care and Public Health, Brighton and Sussex Medical School, University of Sussex, Falmer, UK.; ^6^Department of General Practice, Institute of Health and Society, University of Oslo, Oslo, Norway.; ^7^The Norwegian Organisation for Quality Improvement of Laboratory Examinations (NOKLUS), Haraldsplass Deaconess Hospital, Bergen, Norway.; ^8^Department of Global Public Health and Primary Care, University of Bergen, Bergen, Norway.; ^9^Norwegian Porphyria Centre, Department of Medical Biochemistry and Pharmacology, Haukeland University Hospital, Bergen, Norway.; ^10^Department of Family Medicine, CAPHRI School for Public Health and Primary Care, Maastricht University, Maastricht, The Netherlands.; ^11^Sector Zorg, Zorginstituut Nederland, Diemen, The Netherlands.; ^12^Laboratory for Clinical Chemistry and Haematology, Jeroen Bosch Hospital, ‘s-Hertogenbosch, The Netherlands.; ^13^Cancer Health Services Research, School of Population and Global Health, Faculty of Medicine, Dentistry and Health Sciences, University of Melbourne, Melbourne, VIC, Australia.

**Keywords:** Primary Healthcare, General Practice, Value Network, Rapid Diagnostics, Organization of Care

## Abstract

**Background:** In some countries, such as the Netherlands and Norway, point-of-care testing (POCT) is more widely implemented in general practice compared to countries such as England and Australia. To comprehend what is necessary to realize the benefits of POCT, regarding its integration in primary care, it would be beneficial to have an overview of the structure of healthcare operations and the transactions between stakeholders (also referred to as value networks). The aim of this paper is to identify the current value networks in place applying to POCT implementation at general practices in England, Australia, Norway and the Netherlands and to compare these networks in terms of seven previously published factors that support the successful implementation, sustainability and scale-up of innovations.

**Methods:** The value networks were described based on formal guidelines and standards published by the respective governments, organizational bodies and affiliates. The value network of each country was validated by at least two relevant stakeholders from the respective country.

**Results:** The analysis revealed that the biggest challenge for countries with low POCT uptake was the lack of effective communication between the several organizations involved with POCT as well as the high workload for general practitioners (GPs) aiming to implement POCT. It is observed that countries with a single national authority responsible for POCT have a better uptake as they can govern the task of POCT roll-out and management and reduce the workload for GPs by assisting with set-up, quality control, training and support.

**Conclusion:** Setting up a single national authority may be an effective step towards realizing the full benefits of POCT. Although it is possible for day-to-day operations to fall under the responsibility of the GP, this is only feasible if support and guidance are readily available to ensure that the workload associated with POCT is limited and as low as possible.

## Background

**Key Messages**

** Implications for policy makers**

- Countries should have a dedicated leadership structure in place that governs the task of roll-out and management of healthcare technologies and continuously supports all involved and affected stakeholders.
- Currently, the implementation of point-of-care testing (POCT) is accompanied with higher workloads for clinicians, discouraging them from using innovative technologies.
- Countries should have a dedicated leadership structure or organization responsible for the quality improvement for laboratory services outside the laboratory and hospital. The ministry of health should work together with the appropriate (existing) professional organizations to establish such an organization. There should be improved support structures in place to ensure the responsibilities expected of primary care clinicians are reasonable and they should be provided with set-up assistance, quality control, training and support.

** Implications for the public**

Innovative healthcare technologies can improve patient outcomes when successfully implemented. In some countries, healthcare technologies such as point-of-care testing (POCT) is widely implemented, while in others the uptake is quite slow and lagging. The findings from this research suggest that the biggest barrier to effective wide-scale implementation is a lack of communication between different stakeholders in the healthcare system and a high workload for clinicians aiming to implement POCT. Improved communication and a leadership structure dedicated to the roll-out and management of healthcare innovations such as POCT, could encourage its use by clinicians, and therefore positively contribute to the patient’s experience in the healthcare system.

Diagnostics is an integral part of primary healthcare, as it provides valuable insight to support medical decisions to improve patient outcomes and wellbeing.^1^ Accurate diagnostics can lead to clinical benefits for patients, but also economic benefits for the healthcare system.^2^ For many diseases, both clinicians and patients continue to expect rapid and simple diagnostic tests that can provide results within minutes.^3^ This has led to the development of innovative diagnostics, specifically, easy-to-use analyzers that can be performed at the point of care, more commonly known as point-of-care testing (POCT).^1^

 A point-of-care (POC) test in primary care can be defined as an analytical test that is typically performed during or very close to the time of consultation by a healthcare professional near the point of care instead of a laboratory setting.^4^ The tests often require only a small blood, urine, feces or sputum sample from a patient and can provide test results within a few minutes. This enables a real-time discussion of test results between the general practitioner (GP) and patient during the initial consultations and eliminates the need for a follow-up appointment or telephone discussion.^5^ Subsequently, the consultation process is more convenient for patients and has previously been associated with an increase in patient satisfaction.^6^ POCT has been proven to be cost-effective in areas with limited infrastructure and medical laboratories where it is typically used for easier and faster diagnosis of diseases and infections with high prevalence,^7^ including HIV,^8^ syphilis,^9^ and tuberculosis.^10^ The usefulness of POCT is not only limited to resource-poor settings. It has been shown to be cost-effectiveness in several first world countries for a range of health problems and functions, such as screening for cardiovascular disease,^11^ monitoring patients’ anticoagulant therapy,^12^ and diagnosing respiratory infections^13^ and influenza.^14^ While several studies have shown that POC tests can be cost-effective, ensure high-quality care and even show that outcomes may be better than if patients are monitored by laboratory tests,^15^ access to these tests in some countries is limited.

 Over the past few years, enhanced manufacturing processes and new developments in microchip technology have led to the production of more robust and more accurate POC devices, compared to earlier generations.^16^ Despite these improvements, the implementation of POCT is still predominantly reliant on the active organization and management of clinicians using the tests, including training and quality control.^17^ The implementation of POC tests in primary care varies significantly between countries. A survey published in 2014 looked at the usage of POC tests by primary care clinicians in five countries.^1^ They found that in Australia, for example, the only POC tests that are relatively widely implemented are urine pregnancy tests (68% of respondents), international normalized ratio (INR) tests (48% of respondents), and blood glucose tests (74% of respondents). In comparison, POC tests seem to be much more prevalent in the Netherlands with respondents reporting the use of urine pregnancy tests (94%), urine leucocytes or nitrite tests (96%), blood glucose tests (96%), haemoglobin tests (58%), C-reactive protein tests (48%) and quantitative β-human chorionic gonadotropin tests (22%). The United Kingdom also has high usage of certain tests such as urine pregnancy tests (80%), urine leucocytes or nitrite tests (90%), blood glucose tests (69%) and INR tests (43%). In Norway, 99% of all GPs use POC tests in their practice, with urine strips, blood glucose tests, C-reactive protein tests, haemoglobin tests, INR tests, hemoglobin A1C, urine pregnancy tests, urine albumin-creatinine ratio, streptococc, mononucleose tests and fecal occult blood tests being implemented by more than half of GPs.^18^

 The slow adoption and uptake of certain POC tests have been attributed to several issues, mainly relating to costs and the high workload associated with the implementation. Furthermore, the negative perception of physicians (due to concerns around accuracy, costs and perceived higher workloads) may also contribute to the slow adoption. The recent coronavirus disease 2019 (COVID-19) pandemic has emphasised the significance of rapid, reliable diagnostics and proves that POCT can potentially help reduce the burden on healthcare systems, especially those that are already overwhelmed.^19,20^ It has been shown that the use of COVID-19 POCT has positively affected healthcare providers through improved morale, and reduced worry associated with COVID-19 without disruption of workflow.^21^ The acceptance of COVID-19 POC tests by both healthcare providers and patients will, optimistically, improve the way that POCT is perceived, and contribute to growing adoption rates. The successful implementation of POC tests proven to be cost-effective demands transformation and integration of services across healthcare organizations. There is a need for a better understanding of how POCT fits into the care pathway and how stakeholders influence the implementation.^22^ Furthermore, implementing large-scale changes in a healthcare system successfully is a complicated task. The introduction of POC tests in general practice is not a single event but requires a series of interlinked processes involving several stakeholders with different responsibilities. To comprehend what is necessary to realize the benefits offered by POCT, it is key to understand if differences between health systems can explain different uptake levels. Therefore, it is necessary to have an overview of the actors in the POCT value network, that are involved in the core aspects and the structure of healthcare operations and the transactions between them. A value network can be defined as a network of interconnected and interdependent relationships and activities between actors that determines the way an organization creates and delivers value.^23^ It is merely the conceptualization of the complex relationships between different actors in the healthcare system.

 In this paper, we will use the concepts of value networks to analyse if they can be used to identify factors explaining why some countries have and others have not routinely adopted cost-effective POC tests. This paper maps the value networks in four countries (England, Australia, Norway and the Netherlands) that can explain differences in uptake of POCT by GPs and compares these networks in terms of seven, previously published factors that support the successful implementation, sustainability and scale-up of innovations.

## Methods

###  Identification and Validation of Value Networks

 The methodology applied to identify the value networks is based on a previously published theoretical framework for analyzing a healthcare system as a value network.^23^ For each country, a literature review is conducted to identify each of the respective country’s value network. An initial search of government websites was done for official reports and papers to gain an understanding of each country’s health system as a whole and to identify the stakeholders that play a role in the implementation of POCT. Standards, clinical guidelines, and implementation guides of diagnostics and POC tests within primary care were reviewed to identify the process(es) and requirements of POCT implementation at general practice. Only official documents from governments and organizations affiliated with the government were reviewed. Any journal publications referred to in these documents were included as well if relevant.

 From the full set of information that was gathered during the literature review, the actors that are involved in the value network and the relationships between these actors (the way that actors are connected and communicates) were identified. A visual representation of the value network was developed to comprehend how the different actors connect. The relationships between actors were classified as information, value, and financial transactions or flows.^24^ Information flow refers simply to the movement of information between the different actors, and financial flow encompasses the flow of funds, both receivables (eg, reimbursements) and payables (eg, investments and costs). The value flow refers to the added value between two actors that would drive their Willlingnes to Pay; for example, a POCT can create added value for the GP by providing earlier information of the diagnosis. These flows do not reflect any downstream effects or impacts, such as societal costs or patient benefits. Since the focus of this paper is on primary care, specifically GPs, the presented value networks are from the perspective of the general practice. However, each primary care practice can consist of any number of GPs, and the constructed value network is applicable to either single or multiple GP practices.

###  Health System Perspectives

 If a country has public and private healthcare, the focus of the value network will be on the public system and the mechanism behind the implementation of POCT in the public system.

###  Validation of the Value Networks

 Upon drafting the value networks, each value network was tested for consistency and exchangeability with the core investigators. The value network of each country was validated by the relevant investigators, namely JE and PF for Australia, CPP and HVM for England, SS and TBE for Norway and JTMD and JWLC for the Netherlands. Any uncertainties in the value networks that arose from a misapprehension of the official documents, guidelines, or standards were resolved during the validation process.

###  Comparison of Value Networks

 Upon validation, each of the value networks was summarized in terms of seven key factors that support the successful adoption, implementation, sustainability, spread, and scale-up of service innovations, as identified by Nolte.^25^ A brief description of the seven factors is provided in Supplementary file 1. Details of these factors are published elsewhere.^25^ Based on these seven factors, key differences between the countries were identified and discussed.

## Results

 In all four countries, primary care is typically the first point of contact with the healthcare system and a patient has to be referred by a GP to receive specialist care. Therefore, GPs (as gatekeepers) play an essential part in containing costs. In all countries, if indicated, a consultation with the GP is followed by a sample being collected from the patient for the POC test, either by the GP or the practice assistant/nurse. The test result should be available within a few minutes and the results are discussed with the patient by either the practice assistant/nurse or the GP. GPs may also refer patients to secondary care based on the test results.

###  Australia

**Figure 1:**
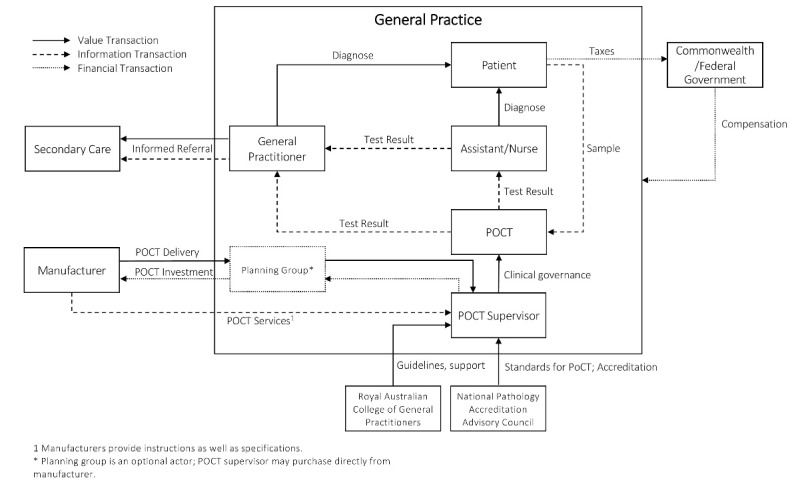
General Practice Point-of-Care Testing Value Network for Australia. Abbreviation: POCT, point-of-care testing.

 A description of the overall health system of Australia is provided in Supplementary file 2. The value network demonstrating the implementation of POCT in general practice for Australia is illustrated in Figure 1. Australia has 31 Primary Health Networks that work directly with GPs and other primary care providers to improve the coordination of care to patients.^26^ In Australia, GPs are typically considered self-employed and part of a practice with an average of four GPs per practice.^27^ For specialist services, a patient can only receive an Medicare Benefits Scheme (MBS) benefit if referred by a GP.

 Australia’s leading professional general practice organization is the Royal Australian College of General Practitioners (RACGP) and provides support to GPs through education, training and developing resources, guidelines and standards that GPs can use to deliver high-quality healthcare.^28^ The RACGP has stated that they believe POCT should be accessible by GPs and covered by MBS.^29^ The Australian College of Rural and Remote Medicine is the professional organisation for many rural GPs. The value network of Australia could potentially be slightly different from a rural perspective.

 There are no mandatory standards or guidelines for GPs to follow when using POCT, and practices are responsible for developing their own quality framework; however, the use of POC tests under these conditions is not covered by MBS. For a GP to be eligible for MBS rebates, the practice must be accredited against the standards for POCT. The standards for POCT (implemented at the general practice) fall under the National Pathology Accreditation Advisory Council (NPAAC), which requires GPs to uphold the same standards as pathology laboratories.^29^ This means that GPs have to follow accreditation measures that were developed for pathology laboratories, which requires each GP to apply to become an approved pathology practitioner and an approved pathology authority. It also requires GPs to register their practice as an accredited pathology laboratory according to Australian Standards administered by the National Association of Testing Authorities, which can be time-consuming and costly as it includes site visits and strenuous administration work. The majority of POCT currently implemented at GPs are conducted without accreditation through National Association of Testing Authorities certified system. This means that there is no governance of the quality for these tests, nor can it be charged to MBS.^30^ As of 2019, less than 20 GPs in Australia using POC tests in their practice have been accredited.^30^

 Currently, there are two sets of standards and guidelines for implementing POCT and ensuring appropriate use at the general practice; one is drafted by NPAAC^31^ and the second by the RACGP.^32^ Both documents set out the requirements when implementing POCT at the GP, such as clinical governance, quality frameworks, training and safety, and waste disposal. Before implementation, practices are required to establish the clinical and diagnostic purpose of the POC tests they wish to implement, based on several reliable sources and provide evidence that the analytical performance of each test method has been evaluated. It is also required to prove that the POC tests will help in meeting the needs of patients in terms of local health infrastructure and other circumstances. The Australasian Association of Clinical Biochemists (AACB) recommends that any healthcare center wishing to implement POCT should set up a planning group consisting of all the staff that will be involved with the use of the POC tests, to share the planning responsibilities (before implementation) required by the standards and guidelines.^30^ This planning group will ideally also decide on which POC tests to purchase and be responsible for the procurement of the devices from the manufacturer. If no planning group is set up, the POCT supervisor (a designated member of the practice who is ultimately responsible for POCT) will be responsible. The guidelines and standards require that each practice has a POCT supervisor (for example, a designated and trained GP, nurse or practice assistant) that oversees the use and management of POCT in the practice, and that this person has a sufficient understanding of both POCT and the POCT standards.^32^

 The POCT supervisor must have completed appropriate POCT training and can delegate some of the responsibilities to another member of the practice, as long as that member has also received POCT training. The supervisor or delegate is also responsible for the quality assurance of POCT within the practice, and must regularly perform and review quality checks, investigate results and performance, and review trends in the quality check results.^31^

 Although the practice should be accredited as a pathology laboratory, the clinicians in the practice using the POC tests (for example, GPs, nurses or assistants) are considered non-laboratory trained personnel. Therefore, the POCT supervisors need to ensure that in addition to training, continuous support is provided to the users. Australia has introduced a regulation that requires POCT manufacturers to supply users with easy to understand instructions as well as specifications that ensures the devices are used correctly. It has been recommended by the AACB that POCT supervisors create active partnerships with manufacturers and other key stakeholders, including the AACB, to ensure continuous support, including additional training and assistance with maintenance and troubleshooting.

###  England

**Figure 2:**
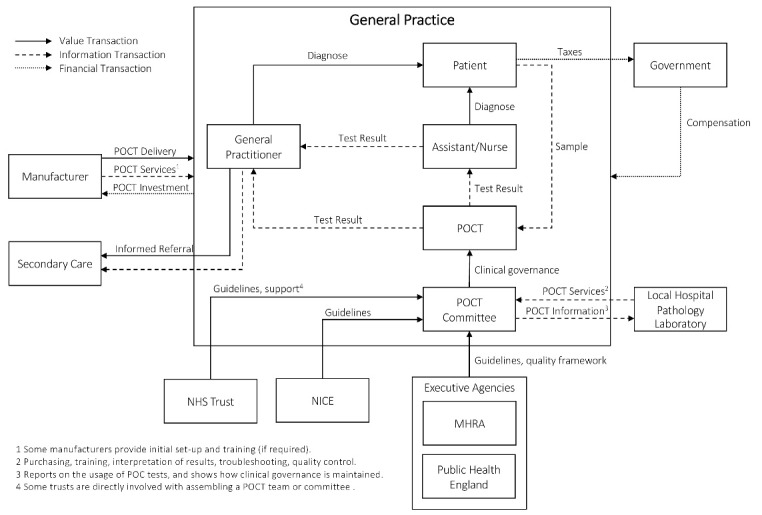
General Practice Point-of-Care Testing Value Network for England. Abbreviations: POCT, point-of-care testing. MHRA, Medicine and Healthcare Products Regulatory Agency; NHS, National Health Service; NICE, National Institute for Health and Care Excellence.

 A description of the overall health system of England is provided in Supplementary file 2. The value network demonstrating the implementation of POCT in general practice for England is illustrated in Figure 2. Within primary care, general practices are owned and managed by an individual or groups of GPs or social enterprises of Community Interest Companies, with a board of directors (who has an APMS contract). They are viewed as independent contractors and are commissioned by Clinical Commissioning Groups (CCGs) to provide services to patients who need it. GPs receive payment from the global sum, to cover the cost of providing routine primary care services to the practice’s registered list of patients. The amount that the practice receives is based on several factors, such as the patients’ age, gender, levels of morbidity and mortality, the area’s index of mean deprivation, the number of patients in nursing and residential homes, patient list turnover and local costs of staff. When a region has specific healthcare needs and priorities, the CCGs (on behalf of National Health Service [NHS] England) may commission community-based services. These include any service that is required to meet the needs of the local population, such as screening for sexually transmitted diseases, weight management, stop smoking programs, etc. Practices can also benefit from financial rewards if certain indicators, as given in the Quality and Outcomes Framework, are met. The Quality and Outcomes Framework is a voluntary incentive scheme, and the majority of practices take part in it, although it is being slimmed down as it seems to lead to further fragmentation of care. The Care Quality Commision do inspections that can lead to practices closing. Furthermore, NHS England sets a prescribing budget for drugs and medication for each of the CCGs on an annual basis. The calculation of the budget is based on several factors, such as the historic spend of CCGs, the local level of deprivation for each GP in the CCG, recent changes in guidelines, new drugs and treatments, prevalence data and population size of the CCG. The CCGs are then responsible for setting a prescribing budget for each general practice within their organization. Typically, CCGs will also develop strategies, such as cost-effective prescribing measures, for GPs to apply. Local commissioners are often GPs.

 Since the GPs are contracted by the NHS, but are not employed by them, they need to follow specific guidelines and quality frameworks. The Medicine and Healthcare Products Regulatory Agency (MHRA) has guided the implementation and management of POCT devices along with a quality framework that should be followed to ensure that all requirements are met. One of the points raised by the MHRA is that a GP interested in adopting POCT within his practice should partake in close collaboration with a local hospital pathology laboratory. The pathology laboratory can give guidance on a diverse range of topics on the implementation and management of POCT. These may include the purchase of devices, quality control, and assessment, training, and safety provisions. For all issues regarding POCT, a close collaboration between the pathology laboratory and the GP using the test is of importance. In most cases, this should be formally defined through, for example, a service level agreement, specifying products, services, practical applications, and responsibilities from the relevant stakeholders. General practices are also strongly encouraged to assemble a POCT committee that represents all immediate stakeholders that will be influenced by the implementation, eg, clinicians, nurses, pharmacists, information technology (IT), finance. These committees should also have a POCT manager that keeps track of their responsibilities towards clinical governance as well as the medico-legal implications of inaccurate results. On top of all their other tasks, this is not a simple consideration. In many practices, the role of the POCT committee is embedded in the local hospital POCT committee with links to the general practice.

 The National Institute for Health and Care Excellence (NICE) is one of the many organizations working with the NHS. They provide the NHS with guidance on how to promote high-quality healthcare and how to prevent and treat illness. Furthermore, they also support healthcare providers and commissioners in improving health outcomes for people using the NHS, public health, and social care services.^33^ The guidance set up by NICE considers both clinical and cost-effectiveness. Technology appraisals performed by NICE is supported by mandate, and NHS England is legally required to provide funding for all medicines and treatments recommended by the institute. The POCT committee should ensure that all guidelines set by the MHRA, NICE, and (if applicable) NHS trusts are adhered to and should also keep track of adherence. Manufacturers can submit the details of their POC test to NICE for consideration and should include sufficient data and analyses proving the accuracy and effectiveness of the device.^34^

###  Norway

**Figure 3:**
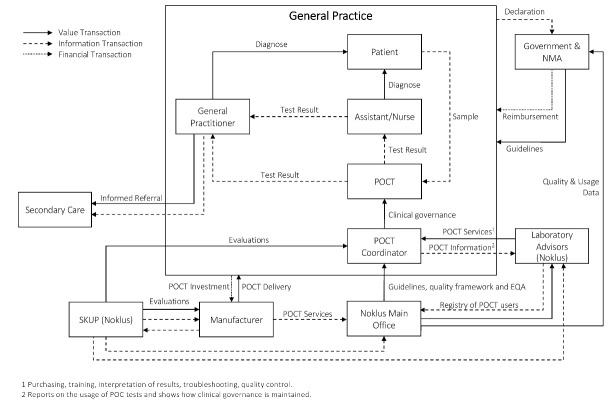
General practice point-of-care testing value network for Norway. Abbreviations: POCT, point-of-care testing; SKUP, Scandinavian evaluation of laboratory equipment for point-of-care testing, NMA, Norwegian Medical Association; EQA, external quality assurance.

 A description of the overall health system of Norway is provided in Supplementary file 2. The value network demonstrating the implementation of POCT in general practice for Norway is illustrated in Figure 3. The majority of GPs are self-employed and part of a practice with two to six physicians. The GPs decide themselves which POCT they offer their patients. The practice forms part of the public system through contracts with the municipalities. GPs receive payment from the municipalities, a fee-for-service from the Norwegian Health Economics Administration and out-of-pocket payments from patients up to a about 250 Euros per year, after which there is no co-payment. The exact payment system is decided on a national level by the Ministry of Health after negotiations with the Norwegian Medical Association (NMA).^35,36^ Approximately 95% of Norwegian physicians are registered members of the NMA, a professional association and a trade union for physicians. The NMA plays an active part in the development of the healthcare system.^37^

 GPs are fairly widespread across the country, but specialist care is typically confined to urban areas.^35^ Most GPs both in urban and rural settings make use of POCT. In 1992, the NMA, the Municipal Association of Local Authorities and the Ministry of Health and Care Services established The Norwegian Quality Improvement of Primary Care Laboratories (Noklus)^38^ to ensure that all POC tests are ordered, performed and interpreted correctly. In 2017, Noklus merged with the Norwegian Clinical Chemistry External Quality Assessment program to form the Norwegian Organization for Quality Improvement of Laboratory Examinations, which focuses on both primary and secondary care. Noklus is a non-profit organization (foundation) that aims to manage and improve the quality of the entire POCT process and covers the entire country. Noklus is also chairing the Scandinavian evaluation of laboratory equipment for POCT (SKUP), that was established in 1997 to improve the quality of POCT throughout Scandanavia. Suppliers and manufacturers of POC tests can pay to have SKUP evaluate their tests. Tests that fail to meet analytical or quality requirements are not recommended to be bought by GPs in Scandanavia.^39^

 Participation in Noklus is not compulsory, yet approximately 99% of all GPs participate willingly.^18^ Noklus is managed by a team consisting of mainly biomedical laboratory scientists but also includes medical doctors, specialists in laboratory medicine, IT programmers, researchers and statisticians. Most of the biomedical scientists are trained as “laboratory advisors” and situated at 22 hospitals across Norway. These advisors supervise and guide the primary care laboratories in their region with regards to quality assurance and all laboratory matters. The advisors are involved with every step of the POCT process, including acquisition, implementation and management.^18^ Their responsibilities include giving individual advice on which POC tests are necessary, advising on maintenance programs, contributing to the protocols set up in terms of test usage, ensuring that quality control programs are followed and evaluated, providing support when problems might arise, and to arrange necessary training for GPs and assistants on the usage and quality control of POC tests. Each advisor has an overview of all POCT users in their region and a total register is maintained by the main office. The professional guidance of the laboratory advisors are done by the main office of Noklus who also runs the external quality assurance (EQA) system for all laboratory users in primary healthcare. Noklus constantly monitor and evaluate the users in terms of quality assurance, usage of tests and any problems that might arise. Noklus also advises the government and the NMA on which tests should be reimbursed, and the majority of tests recommended by Noklus for GPs are reimbursed by the government. Of Noklus’ 3300 participants, 1600 are GP offices. The remainining participants are nursing homes, home care units, oil platforms, prisons etc.

###  The Netherlands

**Figure 4:**
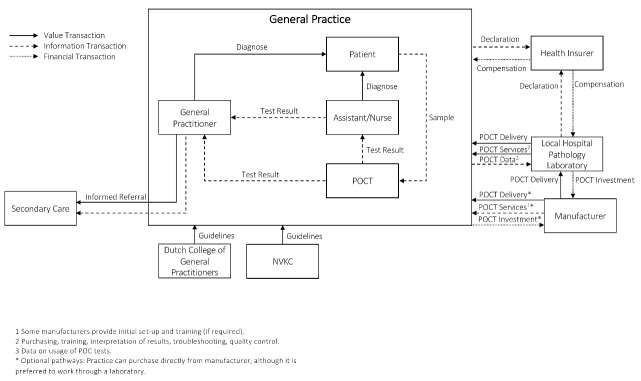
General practice point-of-care testing value network for the Netherlands. Abbreviations: POCT, point-of-care testing; NVKC, Dutch Association for Clinical Chemistry and Laboratory Medicine.

 A description of the overall health system of the Netherlands is provided in Supplementary file 2. The value network demonstrating the implementation of POCT in general practice for the Netherlands is illustrated in Figure 4. In the Netherlands, the GP plays a predominant role, and treats patients for basic health problems and also performs, for example, gynecological or pediatric examinations. Without a referral from your GP for further medical care, such as hospitalization or specialist care, access can be restricted and may not be covered by health insurance. The majority of Dutch GPs work independently or in a partnership, typically in a group practice with two or more GPs. As of 2015, approximately 22% of GPs worked in a single-handed practice.^40^ Many GPs also employ nurses and practice assistants.

 The Dutch college of general practitioners is a scientific association for GPs in the Netherlands. They provide evidence-based guidelines for primary care and also provides education for GPs based on the guidelines.^41^ A guideline directive for the usage of POCT in primary care in the Netherlands^42^ was developed by the Dutch Association for Clinical Chemistry and Laboratory Medicine together with the Dutch college of general practitioners and other organizations. The guidelines are based on international standards and guidelines and provide GPs with recommendations on how to ensure that POCT is used safely and responsibly in practice.

 The guidelines recommend that the practice should consult with a laboratory specialist to ensure that the POC test(s) under consideration is necessary to meet patient demands. Additionally, it is recommended that the practice works with a laboratory specialist throughout the entire implementation process. This includes maintenance of the devices, help with troubleshooting, setting up a quality assurance framework, and annual, or bi-annual verification from the laboratory of POCT performance in terms of diagnostic accuracy, utilization of the tests and whether treatment decisions based on test results are sensible.^42^ Since the guidelines recommend close collaboration between the practice and a laboratory, it is also recommended that all of the POCT usage data (including test results) are recorded in a health information system. This is to enable laboratories to easily assess and compare the quality and utilization of POCT at different GPs.^42^

 The agreements between laboratories and GPs can vary between regions. For example, GPs can purchase POC tests from the manufacturer directly with guidance from laboratories. In most cases, laboratories purchase the POC tests and distribute them to GPs in their region. Reimbursements for the acquisition costs of a POC test or the setup costs of an information system are not available. However, GPs can be reimbursed for the cost price of test kits and for certain (very limited) test devices.^42^ In order for a POC test kit (and certain devices) to be eligible for reimbursement, evidence must show that the POC test is effective. This is a statutory requirement in the Health Insurance Act; specifically, that the test has clinical utility. The National Health Care institute has to issue a positive report on the clinical utility of a test and the Minister of Public Health, Welfare and Sport has to convert that report into a positive decision to reimburse the test and test kits.^43^ For many POC tests the prerequisite of clinical utility has not yet been established in the Netherlands.

###  Comparison of Countries

 The comparison of the four countries and how they support the successful implementation, sustainability and scale-up of POCT are provided in Table.

**Table:** Comparison of Countries

**Factor**	**Australia**	**England**	**Norway**	**The Netherlands**
Leadership and management	Governance mechanisms to provide standards and guidelines for POCT to GPs have been set up. All formal contracts required by these standards are to be organised by the GP. No governance to ensure adherence to these standards. No dedicated leadership structure exists for POCT.	Governance mechanisms to provide standards and guidelines to GPs have been set up; however, there are a few contradictions between the different organizations and bodies. All arrangements required by these guidelines are to be done by the GP. No dedicated leadership structure exists for POCT; it falls under MHRA, PHE, NICE, NHS Trust.	A dedicated POCT organisation has been set up to handle all implementation aspects and provide support to GPs. Provides sustained support and guidance to all participating GPs. Involved with every aspect of the implementation and management process of POC tests.	Governance mechanisms to provide guidelines to GPs have been set up. Separate standards for specifically POCT have not been set up, but instead, standards for in-vitro diagnostics are applied.
Stakeholder involvement	Not all stakeholders are involved in the development of standards and guidelines. Standards require a planning group (consisting of all stakeholders) to be set up by GP to make initial investment decisions. Day-to-day management and clinical governance fall under the responsibility of a single POCT supervisor.	Day-to-day management and clinical governance fall under the responsibility of a POCT committee that has to include several stakeholders. The committee has to set up an agreement with a local pathology laboratory for additional support.	Noklus has a Board consisting of representatives from the Government, the NMA (including representative from GP organisation) and the Norwegian Association for Clinical Chemistry. There is an agreement between Noklus and all regional health authorities. Day-to-day management and clinical governance fall under the responsibility of the GP with continuous guidance from a laboratory advisor from Noklus.	Guidelines are determined by a reasonably wide range of stakeholders. Day-to-day management and clinical governance fall under the responsibility of the GP and laboratory.
Dedicated and ongoing resources	No dedicated resources for POCT. The only way to receive any support is to be registered as a pathology laboratory, which is very expensive and cumbersome.	No dedicated resources for POCT. GPs have to provide their own funding, staff, infrastructure and time. Most GPs would request additional funding from the CCG; however, the CCG has no dedicated funding, so would expect the cost to be covered by savings.	Noklus provides ongoing support. Noklus also offers valuable and low-cost courses for GPs, nurses and other practice assistants to ensure good quality in the use of POCT. Negotiates for reimbursements for tests with the government and the NMA.	Guidelines on implementation are available, but the practice (or local trust of GP practices) itself is responsible for setting up an agreement with a local laboratory to guide implementation. However, funding for the acquisition of a POC test is unavailable.
Effective communication	No data is collected on how GPs follow or experience the guidelines and standards. All communication regarding POC tests is done by or via the POCT supervisor, who is in charge of ensuring clinical governance. No specific communication channels established.	No data is collected on how GPs follow or experience the guidelines and standards. In some cases, guidelines contradict each other. No concrete support is provided on the implementation or whom to report to. In some areas, GPs voluntarily share quality assurance information with hospitals through established channels.	Each county has 2-5 laboratory advisors from Noklus, situated in a local hospital or laboratory whose primary goal is to communicate with GPs in the region to provide support and feedback.	No specific communication channels established. Most laboratories have a POCT coordinator and quality assurance coordinator, who is responsible for quality checks in GP practices. No data is collected on how GPs follow or experience the guidelines and standards, although GPs can request a ‘diagnostic test consultation’ where they receive an analysis of how well the standards are being applied.
Adaption and integration to local context	Standards and guidelines remain the same to everyone, and this is especially restricting in Australia for remote locations. The planning group is responsible for selecting appropriate tests for practice as well as manufacturers.	Local authorities exist that can aid the POCT committee with decisions. The primary responsibility still lies with the committee and the GP to select appropriate tests for practice as well as manufacturers.	Noklus analyzes GPs patients and history to determine which POCT repertoire will be best. The GP decides which POC tests to provide based on the advice from Noklus. Local laboratory advisors evaluate if the implementation can be improved for the area.	GPs are recommended to work with local laboratories to decide on which POC tests will be most useful for their patients.
Ongoing monitoring and feedback	GPs are required to monitor performance themselves. Data collection and management is the responsibility of the practice. If GP is registered at NPAAC, EQA programs deliver peer review of the POCT systems and may monitor performance.	No data collection is done (or required). In some areas, GPs and hospitals voluntarily work together to apply monitoring and feedback processes.	Local laboratory advisors gather data from GPs in their area and send it to the Noklus main office for analysis. They provide ongoing monitoring, calibration, quality checks, and evaluates whether the tests are utilized. Everything is monitored on a web-based database accessible to both GPs and laboratory advisors.	Guidelines recommend a Health Information System to be set up where usage data and results are collected to allow easier assessment. Costs for setting up such systems are not reimbursed. GPs can voluntarily request a ‘diagnostic test consultation’ put in place to support GPs using and interpreting POCT.
Evaluation and demonstration of the effectiveness	MSAC does evaluate POC tests, if a submission is made. GPs are not required to evaluate the effectiveness of POCT since the evidence is available from “other sources” such as suppliers and societies. GPs are responsible themselves to ensure implementation and usage is done according to the manufacturers’ standards and guidelines.	No official evaluation (done by the government) is in place to evaluate the effectiveness of POC tests currently in place at GPs.	Provides quality assessment schemes (EQA) to monitor and improve the usage of devices. SKUP provides evaluations of, eg, analytical quality and user-friendliness.	No official evaluation (done by the government) is in place to evaluate the effectiveness of POC tests currently in place at GPs.

Abbreviations: GP, general practitioner; POCT, point-of-care testing; POC, point-of-care; MHRA, Medicine and Healthcare Products Regulatory Agency; PHE, Public Health England; NICE, National Institute for Health and Care Excellence; NHS, National Health Service; NMA, Norwegian Medical Association; EQA, external quality assurance; NPAAC, National Pathology Accreditation Advisory Council; SKUP, Scandinavian evaluation of laboratory equipment for POCT; MSAC, Medical Services Advisory Committee. Note: The comparison is made in terms of the seven factors published by Nolte.^25^

**Figure 5:**
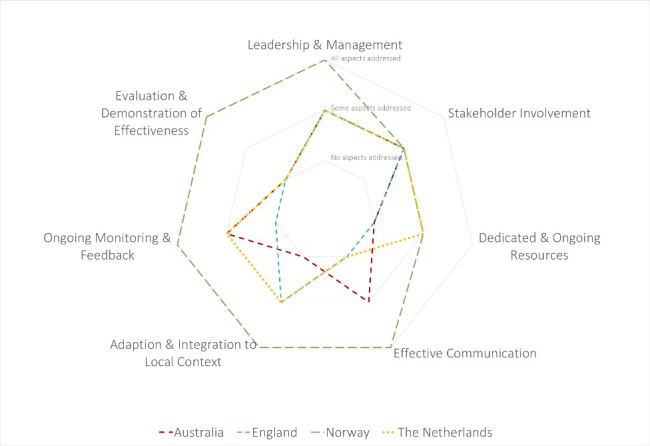
Comparison of the Extent to Which Each Country’s Value Network Addresses Aspects Related to Seven Factors.

 A spider diagram comparing the extent to which each country’s value network addresses aspects related to each of the seven factors is shown in Figure 5. Norway addressed the most aspects of each factor, mainly due to the presence of a single national authority (Noklus) responsible for POCT. Of the four countries, only Norway has a dedicated leadership structure in place that actively supports the implementation and uptake of POCT. In the Netherlands, there are no POCT-specific standards, but instead, POCT falls under the in-vitro diagnostics standards. In Australia, standards and guidelines for pathology laboratories have to be followed, discouraging GPs to follow the typical route to implementation as laid out by the guidelines. Within England’s healthcare system, there is a lack of clear understanding amongst stakeholders of who is responsible for the implementation of innovation.^44^ This makes implementing a POC test in practice seem more complex than that of the Netherlands and Norway, and this complexity may limit the implementation of POC tests. Additional descriptions on the comparison of countries can be found in Supplementary file 2.

## Discussion

 The benefits of POCT can be substantial. However, although POC tests share characteristics they can differ in terms of, for example, the turnaround times, user-friendliness and associated workload. Similarly, the prevalence and incidence of different diseases in different countries will affect how beneficial a test will be in a specific country. Therefore, the exact benefits will depend on the specific POC test and the context it is applied in. Generally, it can reduce unnecessary hospitalizations and referrals to secondary care, while patients benefit from shorter waiting times for results. It has also been shown to increase patient satisfaction.^45^ Even though POCT has been proven to be a valuable tool in primary care, it does not necessarily incentivize GPs directly. Consequently, GPs might not be willing to take on an additional (high) workload or spend money to implement POCT in their practice. Therefore, support and guidance are necessary to encourage GPs to implement POCT in order to realize the benefits for patients and the health system. A dedicated leadership structure (such as Noklus) should be in place that actively supports the implementation and uptake of POCT. It seems likely that the organization and management of POCT would be more efficient if a separate team or organization, set up by the government, is responsible for all matters related to POCT. Each of the four countries, with their differences in health systems, has different principles when it comes to establishing a dedicated leadership structure or organization to facilitate implementation. As there are differences, it is not possible to define one generalizable approach, and thus, the ministry of health should work together with the appropriate (existing) professional organizations, such as medical associations and professional societies for GPs, laboratory professionals and healthcare providers, to set up a system of quality improvement for laboratory services outside the laboratory and hospital. Clear, well-defined guidelines and standards should be in place that are specific to POCT. In the countries where POCT falls under the umbrella of other guidelines (such as in-vitro diagnostics or pathology guidelines), GPs can be discouraged from following the required route to implementation as it is too complex and cumbersome.

 Such a POCT team should be involved throughout the implementation process, providing guidance to GPs on executing all aspects set out by the guidelines and standards. Furthermore, all stakeholders that would be affected by the implementation of POCT, including patients, manufacturers, and GPs, should be involved in setting up the standards and guidelines to improve commitment. One of the most significant barriers to POCT implementation for GPs is the high workload associated with setting up POCT in a practice. It is vital that GPs be part of setting up guidelines and standards to ensure enough support is provided and that the responsibilities expected of the GPs are reasonable.

 Dedicated and ongoing resources is a factor that is especially important for the implementation of POCT. Financial resources can improve the uptake of POCT if it allows GPs to adopt POCT within their practice without additional cost. In the case of Norway, the NMA in cooperation with Noklus negotiates reimbursements from the government for financial support, while in the Netherlands, GPs can make arrangements with laboratories. GPs could, potentially, also be offered a financial incentive to use POCT by returning the downstream cost savings realised in the healthcare system. Ongoing support for GPs is also a vital resource to reduce the workload and encourage implementation. Support during the initial implementation process is required to help GPs select a POCT repertoire that suits local needs. Ongoing monitoring and feedback are required to identify any opportunities for improvement within a practice. The guidelines and governing team should clearly provide GPs with instructions and support to set up a data collection system to collect and assess the performance of tests systematically. This will also simplify the process of quality assurance and evaluating the effectiveness of the POC tests in place at GPs.

 Although the value network of one country cannot simply be transferred to another country, the results remain important in understanding the critical factors behind the successful implementation of POCT. These value networks help to comprehend what the value is of using a POC test, where this value is delivered, and which stakeholders are driving the value generation. These aspects, together with the strengths and weaknesses observed in the value networks, will be helpful when it comes to strategic thinking and can be used as a starting block to set up rigorous implementation plans and roll-out plans. One limitation of this paper is that the results are not applicable to low- and middle-income countries (LMICs). This is mainly due to the fact that the implementation of POC tests in these countries are mostly governed by both the healthcare system and by the World Health Organization (WHO) and donors.^46^ The value networks in these countries will, therefore, be very different than those in this paper, and will be hard to compare. Nonetheless, there are lessons that can be learned from the value networks presented in this paper and potentially from the value networks in LMICs. Future research should aim to identify the value networks in place in LMICs and investigate how comparable it is to those of high-income countries or what specific innovation and/or business models would apply.

 It is expected that the global POC diagnostics market will reach $40.50 billion by 2022.^47^ However, efforts in developing POC tests and identifying cost-effective POC tests are wasted when their benefits are not realized. From the value networks identified in this paper, it is evident that differences exist in the organization of care between countries, which quite likely cause part of the observed differences in POCT adoption. The comparison of the value networks of different countries is useful in determining how countries can move forward in realizing the benefits of POCT, especially where adoption is low. It is observed that if a single national authority is responsible for POCT, the uptake of POCT may improve since they can govern the task of roll-out and management, and reduce the workload for GP’s by assisting with set-up, quality control, training and support. However, this might be predicated on the governance of a country. For example, allocating a single national POCT authority, while feasible, could work differently in a federation (such as Australia) regarding establishing and delivering a value network for POCT. Although it is possible for day-to-day operations to fall under the responsibility of the GP, this is only feasible if support and guidance are readily available to ensure that the workload associated with POCT is limited and as low as possible. Bringing about the necessary changes and integration can be complex and time-consuming, but it is nonetheless feasible, given the example of Norway. Future quantitative analysis could indicate the magnitude of opportunity loss caused by a lack of POCT adoption as incentive for initiating these necessary changes.

## Ethical issues

 An ethical approval was not necessary, since no human data was used. Only guidelines and reports that are openly published by the relevant governments and organizations were used.

## Competing interests

 Authors declare that they have no competing interests.

## Authors’ contributions

 DL, HK, RK, and MJI provided input into the conception and study design. DL drafted the manuscript with input from HK, RK, and MJI. HK, RK, MJI, JDE, PF, CPP, HVM, TBE, SS, JWLC, and JTMD provided revisions to the manuscript.

## Supplementary files

Supplementary file 1. Description of Factors.

Supplementary file 2. Additional Description and Comparison of Each Country’s Health System.
