# Supplementary material for: How to Realize the Benefits of Point-of-Care Testing at the General Practice: A Comparison of Four High-Income Countries
Source: Int J Health Policy Manag. 2021 Oct 13;11(10):2248–60. doi: 10.34172/ijhpm.2021.143 (PMC9808289; doi:10.34172/ijhpm.2021.143)
Supplement: Supplementary file 1 — Description of Factors. [file ijhpm-11-2248-s001.pdf]

Article title: How to Realize the Benefits of Point-of-Care Testing at the General Practice: A Comparison of Four High-Income Countries

Journal name: International Journal of Health Policy and Management (IJHPM)

Authors' information: Deon Lingervelder[1]*, Hendrik Koffijberg[1], Jon D. Emery[2], Paul Fennessy[3], Christopher P. Price[4], Harm van Marwijk[5], Torunn B. Eide[6], Sverre Sandberg[7,8,9], Jochen W.L. Cals[10], Joke T.M. Derksen[11], Ron Kusters[1,12], Maarten J. IJzerman[1,13]

[1]Health Technology and Services Research Department, Technical Medical Centre, University of Twente, Enschede, The Netherlands.

[2]Department of General Practice and Centre for Cancer Research, University of Melbourne, Melbourne, VIC, Australia.

[3]Department of Health & Human Services, State Government of Victoria, Melbourne, VIC, Australia.

[4]Nuffield Department of Primary Care Health Sciences, University of Oxford, Oxford, UK.

[5]Department of Primary Care and Public Health, Brighton and Sussex Medical School, University of Sussex, Falmer, UK.

[6]Department of General Practice, Institute of Health and Society, University of Oslo, Oslo, Norway.

[7]The Norwegian Organisation for Quality Improvement of Laboratory Examinations (NOKLUS), Haraldsplass Deaconess Hospital, Bergen, Norway.

[8]Department of Global Public Health and Primary Care, University of Bergen, Bergen, Norway.

[9]Norwegian Porphyria Centre, Department of Medical Biochemistry and Pharmacology, Haukeland University Hospital, Bergen, Norway.

[10]Department of Family Medicine, CAPHRI School for Public Health and Primary Care, Maastricht University, Maastricht, The Netherlands.

[11]Sector Zorg, Zorginstituut Nederland, Diemen, The Netherlands.

[12]Laboratory for Clinical Chemistry and Haematology, Jeroen Bosch Hospital, 's-Hertogenbosch, The Netherlands.

[13]Cancer Health Services Research, School of Population and Global Health, Faculty of Medicine, Dentistry and Health Sciences, University of Melbourne, Melbourne, VIC, Australia.

(*Corresponding author: d.lingervelder@utwente.nl)

Supplementary file 1. Description of Factors

Each of the value networks was summarized in terms of seven key factors that support the successful adoption, implementation, sustainability, spread, and scale-up of service innovations, as identified by Nolte.[1] A brief description of the seven factors is provided in Table S1. Details of these factors are published elsewhere.[1]

Table S1. Description of the Seven Factors Outlined by Nolte [1].

**Factor**	**Description**
Leadership and management	Support from all tiers of leadership and management in the healthcare system, including clear goals and guidelines.
Stakeholder involvement	Widespread stakeholder involvement during the implementation process, including developing structures and guidelines.
Dedicated and ongoing resources	Funding and support throughout the implementation process to guide the design and implementation, as well as for staff and capacity building.
Communication	Effective communication across and between all organizations involved with the implementation and definite appointment of roles responsibilities.
Adaption and integration to local context	For sustainability, the implementation has to be adapted to the local needs and possibly integration with existing policies.
Ongoing monitoring and feedback	Assessing performance and identifying areas for improvement through data collection at each GP.
Evaluation and demonstration of effectiveness	Assessing effectiveness and utilization through quality checks and monitoring.

References

1. Nolte E. How do we ensure that innovation in health service delivery and organization is implemented, sustained and spread? *WHO Reg Off Eur Policy Br*. 2018.
