# Supplementary material for: How to Realize the Benefits of Point-of-Care Testing at the General Practice: A Comparison of Four High-Income Countries
Source: Int J Health Policy Manag. 2021 Oct 13;11(10):2248–60. doi: 10.34172/ijhpm.2021.143 (PMC9808289; doi:10.34172/ijhpm.2021.143)
Supplement: Supplementary file 2 — Additional Description and Comparison of Each Country’s Health System. [file ijhpm-11-2248-s002.pdf]

Article title: How to Realize the Benefits of Point-of-Care Testing at the General Practice: A Comparison of Four High-Income Countries

Journal name: International Journal of Health Policy and Management (IJHPM)

Authors' information: Deon Lingervelder[1]*, Hendrik Koffijberg[1], Jon D. Emery[2], Paul Fennessy[3], Christopher P. Price[4], Harm van Marwijk[5], Torunn B. Eide[6], Sverre Sandberg[7,8,9], Jochen W.L. Cals[10], Joke T.M. Derksen[11], Ron Kusters[1,12], Maarten J. IJzerman[1,13]

[1]Health Technology and Services Research Department, Technical Medical Centre, University of Twente, Enschede, The Netherlands.

[2]Department of General Practice and Centre for Cancer Research, University of Melbourne, Melbourne, VIC, Australia.

[3]Department of Health & Human Services, State Government of Victoria, Melbourne, VIC, Australia.

[4]Nuffield Department of Primary Care Health Sciences, University of Oxford, Oxford, UK.

[5]Department of Primary Care and Public Health, Brighton and Sussex Medical School, University of Sussex, Falmer, UK.

[6]Department of General Practice, Institute of Health and Society, University of Oslo, Oslo, Norway.

[7]The Norwegian Organisation for Quality Improvement of Laboratory Examinations (NOKLUS), Haraldsplass Deaconess Hospital, Bergen, Norway.

[8]Department of Global Public Health and Primary Care, University of Bergen, Bergen, Norway.

[9]Norwegian Porphyria Centre, Department of Medical Biochemistry and Pharmacology, Haukeland University Hospital, Bergen, Norway.

[10]Department of Family Medicine, CAPHRI School for Public Health and Primary Care, Maastricht University, Maastricht, The Netherlands.

[11]Sector Zorg, Zorginstituut Nederland, Diemen, The Netherlands.

[12]Laboratory for Clinical Chemistry and Haematology, Jeroen Bosch Hospital, 's-Hertogenbosch, The Netherlands.

[13]Cancer Health Services Research, School of Population and Global Health, Faculty of Medicine, Dentistry and Health Sciences, University of Melbourne, Melbourne, VIC, Australia.

(*Corresponding author: d.lingervelder@utwente.nl)

Supplementary file 2. Additional Description and Comparison of Each Country's

Health System

1.1. Description of Australia's Health System

In Australia, universal health care falls under the shared responsibility of three levels of government, namely, federal, state or territory, and local. The federal government plays a limited role in direct service delivery and is mostly responsible for the funding and (indirect) support to the states and their healthcare professionals. The federal government is the source of funding for primary care providers through the Medicare Benefits Scheme (MBS) and the Pharmaceutical Benefits Scheme (PBS) [1]. States or territories fund public hospitals, mental health, dental health and a limited range of primary care services, in particular the public community-based and allied health services, etc. The local governments provide certain co-republic health functions and some limited preventative health programs. This includes, for example, waste disposal, water supply, smoking cessation and weight loss programs [2].

Furthermore, the healthcare system has both a public and private system counterpart. Patients have access to healthcare through one of the systems or a mix of both. Both systems comprise of several components, such as hospitals, community services and other health organizations. The private system healthcare services are owned and operated privately [1,3]. These services are licensed and regulated by governments and funded by both government and private entities, including private health insurance (paid by patients), private organizations and private funding. The public system healthcare services are owned and operated solely by the state and territory governments [2]. Services in the public system are funded by local and state and federal governments, and healthcare access is covered by the MBS for free or at a lower cost to patients [2,3]. Patients that makes use of private insurance can also access all of the services, but the MBS only covers 75% - 85% of the cost, depending on the service. The remaining costs has to be paid by the private insurer or the patient themselves. MBS is funded, in part, from general taxes and free to all Australian citizens and residents with a permanent visa. It covers the costs to patients for public hospital services in full and some or all of the costs for other health services, such as services provided by a GP or specialist. The PBS provides subsidies to patients for pharmaceuticals that are approved for cost-effectiveness by the Pharmaceutical Benefits Advisory Committee (PBAC) [2].

1.2. Description of England's Health System

The National Health Service (NHS) in the United Kingdom is paid from national insurance and free to all permanent residents at the point of use. England, Northern Ireland, Scotland and Wales each have their own, slightly different health care system in place that is funded and administered by separate 'devolved' governments, each with some individual policies and priorities. In terms of population, England is by far the largest and will be the focus of this study. The UK government is responsible for top-level priority setting and determines the budget for the NHS. Through the Department of Health, they oversee the healthcare system and set the overall policy, strategy, and funds. There is a range of organizations that the Department of Health provides funding to, but the most significant portion of the budget goes to NHS England. NHS England oversees the commissioning: the planning and buying of healthcare services. They pass the majority of their money (about 66% of the budget) to 135 clinical commissioning groups (CCGs), situated across England [4]. The CCGs (in partnership with Local Authorities) are responsible for identifying the healthcare needs of their area and for commissioning healthcare services for the residents of that area accordingly. They can commission services from any organization that provides care, such as GPs, community services, ambulances, etc. The CCGs have joint responsibility with NHS England; however, they can opt to take on full accountability for commissioning medical care services.

From 1 April 2019, NHS England is working together with NHS Improvement as a single organization. Since specialists (consultants) are all employed by the NHS (and GPs are not), the system does not facilitate referrals or seamless pathways. This is different from countries where consultants' incomes depend largely on accepting referrals and on a good relationship with GPs. At the inception of the NHS, it was considered too costly to bring GPs in. Some consultants do extra private work. NHS Improvement is responsible for administering NHS trusts and ensuring that trusts work efficiently and cost-effective. Hospital and specialist care are primarily delivered by NHS foundation trusts and NHS trusts, although they can sometimes be directly involved with some primary care services as well in areas of great need or when no local GPs are avaiable. Most consultants working in primary care are working for private companies who have contracts commissioned by CCGs, as this is more cost effective than out patients in hospitals. Typically, an NHS trust provides care to a specific region or serves as a specialized function (e.g., an ambulance service). It is possible for one region to have several trusts working towards different aspects of healthcare. They work closely with CCGs in meeting the local needs. NHS England is currently developing a more integrated

approach to services. There is an initial tranche of 14 integrated care services across England with the intention of complete coverage in 2020/21. The integrated care services are driving practices to merge together, thereby creating an environment more amenable to local testing such as POCT [5].

The Department of Health also provides funding to Public Health England and the Medicine and Healthcare Products Regulatory Agency (MHRA). Public Health England aims to serve the government, NHS, industry, and the public by providing evidence-based research, support, and expertise. They also run public health campaigns. The MHRA is in charge of regulating medicines, medical devices, and blood components for transfusion in the UK. Local authorities are responsible for public health, which includes a wide range of healthcare services, including education, transportation, waste disposal, environmental health, and many more. Since public health is their primary concern, they work in close collaboration with CCGs to ensure that the healthcare needs of the region are being met efficiently. GPs are independent contractors with an NHS contract to provide services to a population of patients. There are currently three main types of core contract: General Medical Services (GMS), Personal Medical Services (PMS) and Alternative Provider Medical Services (APMS). GMS is the contract agreed nationally and stipulates essential services to be provided through several types of GP contracts. PMS contracts are phased out. Private healthcare providers who manage walk-in clinics, have a five year APMS contract [6].

1.3. Description of Norway's Health System

Norway has a public healthcare system, financed by taxation and patient co-payments. Healthcare falls under the responsibility of three levels of government, namely the central State, regional health authorities (RHAs), and local municipalities [7,8]. Healthcare policies, legislation, and funding is managed centrally by the government and its ministries (Central state), while primary healthcare provision is decentralized and falls under the responsibility of local municipalities. The ministry owns and funds most of the hospitals in the country and is responsible for specialist care, through its four RHAs [9]. There are a few private hospitals, some of which are funded through contracts with the public healthcare system [8]. All of Norway's inhabitants are covered by the National Insurance Scheme (NIS) that is managed by the Norwegian Health Economics Administration (Helfo). Private health insurance is also available from for-profit insurers, although only about 9% of the population has some kind of

private insurance [7]. Pricing and reimbursement decisions are predominantly made on a national level, with separate systems for primary care and specialist care. The Norwegian Health Economics Administration (Helfo) is responsible for the actual reimbursement of all pharmaceuticals, devices and services covered by the NIS [9].

1.4. Description of the Netherlands' Health System

In the Netherlands, healthcare priorities, legislation and monitoring of access, quality and costs fall under the responsibility of the national government. In addition to general laws and acts set up by the government, the foundation of the healthcare system is formed by four acts, namely, the Health Insurance Act, the Long-Term Care Act, the Social Support Act, and the Youth Act. The Long-Term Care Act is there to aid the most vulnerable groups, specifically patients that require permanent care. It falls under the responsibility of the government, but there are several organizations involved with its implementation. Both the Social Support Act and the Youth Act falls under the responsibility of municipalities and local authorities. Under the Social Support Act, people who struggle to participate in society or cannot care for themselves, are provided for and supported by the municipality and local authorities. The Youth Act was introduced for the support and care of children and adolescents.

The Health Insurance Act is implemented by healthcare insurers and providers. Under the Health Insurance act, it is mandatory for everyone who lives or works in the Netherlands to have basic health insurance. Therefore, insurers are obliged to accept everyone that applies for the basic insurance and have to charge them the same premium. Insurers are allowed to try and attract customers by offering lower prices. However, they are not allowed to ask a higher premium for people with, for example, a certain disease. In addition to the nominal premium, everybody pays an income-related contribution for the standard package that is remitted to the health insurance fund by the employer. Basic health insurance covers medical care, medicines and hospitalization. All insurers have to offer the same standard package, although it may have different premiums and may be extended with additional advantages. Additional insurance for eye care, dental care or physiotherapy can be added at an extra cost. In addition to the premiums set by the health insurer, every insured person has an annual own-contribution, which is the amount that is to be paid by each person themselves, before the insurer covers any medical costs. This does not apply to all medical care, for example, GP services or maternity care, but POCT and laboratory tests requested in primary care have to

be paid by the patient from their annual own-contribution with a yearly determined maximum threshold.

1.5. Comparison of Country Performance

Stakeholder involvement is crucial to ensure that everyone who will be impacted by POCT is on board with the implementation process. For England and the Netherlands, the day-to-day management of POCT in a practice falls under the responsibility of the GP with support from a local laboratory (selected by the GP) with no concrete outside support. Only in Norway is there a dedicated advisor appointed by Noklus for each region to provide ongoing support and guidance to practices using POCT. In Australia, GPs who want to make use of POCT should register as a pathology laboratory, and consequently receives no support from an external laboratory.

Australia and England fall short in terms of dedicated and ongoing resources since no dedicated resources for POCT are available for GPs. In terms of financial resources, GPs in Australia could be eligible for some reimbursement, but only if the practice is registered as a pathology laboratory which is very expensive and cumbersome. In the Netherlands, funding is available for GPs to appoint a practice nurse or assistant, which is useful when using POCT. For Australia, England and the Netherlands, GPs have to take the initiative to adopt POCT in their practice and follow implementation procedures as laid out by the guidelines, without support. In Norway, there is ongoing support from Noklus and each region has a laboratory advisor. Noklus and the NMA also negotiate reimbursements from the government for financial support, based on the evaluations from SKUP.

As is clear from the value network of each country, there are several organizations involved with the implementation of POCT at a practice. In Norway, Noklus plays a critical part in ensuring effective communication, with a laboratory advisor acting as an intermediary for GPs and all other organizations. Noklus mainly works to ensure quality of the POCT, while it remains the responsibility of the general practice to ensure that the practice is properly run. The other countries do not have a dedicated communication channel established, and it is the practice’s responsibility to ensure that guidelines and standards are being followed and that communication between the practice and a local laboratory (in the case of the Netherlands and England) takes place effectively.

England and the Netherlands both have some local adaption, with local authorities in England and local laboratories in the Netherlands helping POCT committees and GPs with decisions. In Australia, there is no support to adapt to a local context. This is especially problematic, seeing as the several remote areas that would benefit the most from POCT are held accountable under the same rules as GPs in urban areas. In Norway, Noklus is actively involved to help GPs implement a POCT repertoire that is specific to the practice's needs.

Setting up data collection systems to collect and assess performance systematically and to identify any opportunities for improvement. In Australia and England, no data collection is officially required, and any data collection and monitoring falls under the responsibility of the practice. If a practice is accredited in Australia, there is some performance monitoring. Guidelines in the Netherlands do recommend that an information system is set up between the practice and the laboratory to ensure the laboratory can monitor performance. In Norway, the local laboratory advisors gather data from the POCT of GPs to assess the quality of the tests, and also sends it to the Noklus main office for further analysis.

Evaluation and demonstration of the effectiveness of POCT implementation at the practice are only addressed in Norway, where Noklus provides quality assessment schemes. For the remaining three countries, there is no official evaluation in place to demonstrate the effectiveness of POCT. The countries do perform some health economic evaluations of devices, but they do not evaluate the effectiveness of the POC tests at each practice.

References

1. Glover L. Australia : International Health Care System Profiles. https://international.commonwealthfund.org/countries/australia/. Published 2017. Accessed March 23, 2020.

2. Australian Institute of Health and Welfare. How does Australia ' s health system work? *Aust Heal*. 2016;(15):1-13.

3. Australian Government Department of Health. The Australian health system. https://www.health.gov.au/about-us/the-australian-health-system. Published 2019. Accessed April 6, 2020.

4. NHS England, NHS Improvement. *Fair Shares: A Guide to NHS Allocations*.; 2017.

5. National Health Service. *Designing Integrated Care Systems (ICSs) in England: An Overview on the Arrangements Needed to Build Strong Health and Care Systems*

across the Country.; 2019. https://www.england.nhs.uk/wp-content/uploads/2019/06/designing-integrated-care-systems-in-england.pdf.

6. Addicott R, Ham C. *Commissioning and Funding General Practice Making the Case for Family Care Networks Authors*.; 2014.

7. Lindahl AK. Norway : International Health Care System Profiles. https://international.commonwealthfund.org/countries/norway/. Published 2017. Accessed March 30, 2020.

8. Norwegian Medicines Agency. The Norwegian health care system and pharmaceutical system. https://legemiddelverket.no/english/about-us/the-norwegian-health-care-system-and-pharmaceutical-system. Published 2016. Accessed March 20, 2020.

9. Festøy H, Hagen C, Weise N. *PPRI Pharma Profile: Norway*.; 2018.
